# Characterization of hyaluronan and TSG-6 in skin scarring: differential distribution in keloid scars, normal scars and unscarred skin

**DOI:** 10.1111/j.1468-3083.2010.03792.x

**Published:** 2011-03

**Authors:** KT Tan, DA McGrouther, AJ Day, CM Milner, A Bayat

**Affiliations:** † Department of Plastic & Reconstructive Surgery, University Hospital of South Manchester NHS Foundation Trust; ‡ Plastic & Reconstructive Surgery Research, Manchester Interdisciplinary Biocentre,; § Wellcome Trust Centre for Cell-Matrix Research, Faculty of Life Sciences, University of Manchester,Manchester, United Kingdom

**Keywords:** hyaluronan, inter-α-inhibitor, keloid, scarring, TSG-6

## Abstract

**Background** Hyaluronan (HA) is a major component of the extracellular matrix (ECM) with increased synthesis during tissue repair. Tumour necrosis factor-stimulated gene-6 (TSG-6) is known to catalyze the covalent transfer of heavy chains (HC1 and HC2) from inter-α-inhibitor (IαI) onto HA, and resultant HC•HA complexes have been implicated in physiological and pathological processes related to remodelling and inflammation.

**Objective** The aims of this study were to determine the expression of HA, TSG-6 and the IαI polypeptides in unscarred skin, normal scars and keloid scars.

**Methods** Formalin-fixed paraffin-embedded sections of unscarred skin, normal scars and keloid scars were prepared from patient samples collected during scar revision surgery. Haematoxylin and eosin, as well as immunofluorescent staining for HA, TSG-6 and the three polypeptide chains of IαI (i.e. HC1, HC2 and bikunin) were performed.

**Results** All skin types stained positive for TSG-6, HC1, HC2 and bikunin, associated with keratinocytes, fibroblasts and skin appendages all in close proximity to HA. Keloid lesions showed altered HA organization patterns compared with unscarred skin and normal scars. TSG-6 staining was significantly more intense in the epidermis compared with the dermis of all sample types. There was a significant reduction in TSG-6 levels within keloid lesions compared with the dermis of unscarred skin (*P* = 0.017).

**Conclusion** TSG-6 is expressed in unscarred skin, where its close association with HA and IαI could give rise to TSG-6-mediated HC•HA formation within this tissue. A reduction in the beneficial effects of TSG-6, caused by diminished protein levels in keloid lesions, could contribute to this abnormal scarring process.

## Conflict of interest

None.

## Introduction

Cutaneous scarring can be defined as a macroscopic disturbance of the normal skin structure and function, following wound healing.^1^ Keloid scars are abnormally raised cutaneous scars that extend beyond the boundaries of original injury.^2–6^ Various factors including genetic background, fibroblast phenotype, growth factor regulation and extracellular matrix (ECM) composition are implicated in the pathogenesis of keloid formation.^7^ The results of genetic linkage analyses are indicative of keloid susceptibility loci within the chromosome regions 2q23, 7p11 and 14q22–23.^2,3,8^

The Bioimaging Facility microscopes used in this study were purchased with grants from the BBSRC, the Wellcome Trust and the University of Manchester Strategic Fund. Work in AJD/CMM’s laboratory was supported by the Medical Research Council (grant 76445). AB acknowledges the support of NIHR (UK). CMM was supported by Arthritis Research UK (grant 16539).

Hyaluronan or hyaluronic acid (HA) is a high molecular weight glycosaminoglycan that has diverse biological functions (including mediation of cell–cell and cell–matrix interactions) and is a major component of the ECM.^9^ HA plays important roles in both the inflammatory and granulation stages of cutaneous wound repair.^10,11^ It acts to both promote (e.g. via cell recruitment) and limit (e.g. through scavenging of reactive oxygen species) inflammation, while the HA-rich nature of granulation tissue is thought to facilitate cell migration/proliferation and angiogenesis, as well as being structurally important.^11^

Tumour necrosis factor-stimulated gene-6 (TSG-6)^12^ maps to chromosome 2q23.3,^12–14^ i.e. within one of the chromosomal regions suggested to contain a susceptibility locus for keloid scarring.^2^ TSG-6 protein is produced and secreted by many different cell types in response to various stimuli, particularly in physiological and pathological contexts associated with inflammation and tissue remodelling.^14,15^ Inter-α-inhibitor (IαI) belongs to a family of serine protease inhibitors that consist of one or more heavy chains (HC) linked to the protein bikunin by a chondroitin sulphate moiety.^16–18^ This abundant serum protein can enter tissues, e.g. because of increased vascular permeability, during inflammation.

When TSG-6, HA and IαI co-localize (e.g. at inflammatory sites), TSG-6 can catalyze the transfer of HCs from IαI onto HA via two sequential transesterification reactions, resulting in the generation of covalent HC•HA complexes.^16–18^ HC•HA is more prone to cross-linking than unmodified HA,^19^ where this is thought to involve non-covalent HC/HC interactions.^20^ For example, TSG-6-mediated HC•HA formation is essential for the correct organization of the HA-rich matrix that forms around the oocyte prior to ovulation.^21^ HA networks formed through cross-linking may also act as a scaffold for matrix regeneration during tissue repair.^20^ HC•HA has greater avidity than free HA for CD44, thereby potentially increasing leucocyte adhesion to the vascular endothelium at inflammatory sites.^22^

### Aims of the study

The aims of this study were to determine the localizations of HA, TSG-6 and the IαI polypeptides in unscarred skin, normal scar and keloid scar tissues to provide an indication of their involvement in skin scarring and keloid scar formation.

## Materials and methods

### Patient selection and tissue collection

Participants were recruited from patients undergoing scar revision surgery at the Department of Plastic and Reconstructive Surgery at the University Hospital of South Manchester, United Kingdom (Table 1). A total of 19 specimens, consisting of seven keloid scars (patient age 16–45 years; mean 27.3 years) and six normal (non-hypertrophic, non-keloid) scars (patient age 17–59; mean 34.3 years) were collected. Six unscarred skin specimens (patient age 17–49 years; mean 37.5 years) were collected from tissue excised from regions adjacent to keloid and normal scars. This study received full approval from the Research Ethics Committee.

**Table 1:** Study participants and type of specimens collected

Age	Gender	Ethnicity	Site of scar	Cause of scar	Scar type
45	Male	Mixed	Scalp	Shaving	Keloid scar
16	Male	Caucasian	Back of the ear	Accident	Keloid scar
26	Female	Caucasian	Chest	Chickenpox	Keloid scar
20	Female	Caucasian	Back	Surgery	Keloid scar
40	Male	Black African	Abdomen	Laceration	Keloid scar
19	Male	Caucasian	Face	Accident	Keloid scar
25	Female	Black African	Chest	Boil	Keloid scar
41	Female	Caucasian	Abdomen	Surgery	Normal scar
49	Female	Caucasian	Abdomen	Surgery	Normal scar
17	Female	Caucasian	Arm	Burn	Normal scar
23	Female	Caucasian	Abdomen	Surgery	Normal scar
17	Female	Caucasian	Hip	Surgery	Normal scar
59	Female	Black Caribbean	Lip	Accident	Normal scar

### Haematoxylin and eosin staining

Tissue was fixed in formalin for 48 h before paraffin-embedding and sectioning at a thickness of 4 μm. Tissue sections were then subjected to haematoxylin and eosin (H&E) staining, using Harris haematoxylin (Surgipath, Richmond, USA), 1% (v/v) acid alcohol [1% (v/v) concentrated hydrochloric acid in 70% (v/v) ethanol] and 0.1% (v/v) eosin (Surgipath), using a Shandon Linistain™ GLX Linear Stainer (Thermo Scientific, Cheshire, UK) with a standard H&E staining protocol. H&E images were characterized on the basis of morphology by a histopathologist.

**Figure 1:**
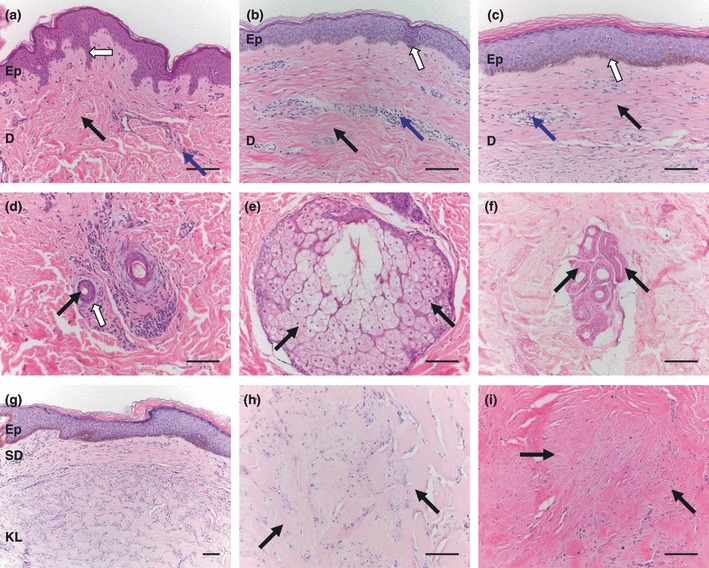
Haematoxylin and eosin (H&E) staining. Unscarred skin (a) with an undulating dermal-epidermal junction (white arrow) and haphazardly arranged dermal collagen bundles (black arrow). A normal scar (b) with a flattened epidermis (white arrow) and thin strand-like collagen bundles (black arrow) parallel to the epidermis. A keloid scar (c) with flattened epidermis (white arrow) and thin, parallel strand-like collagen bundles (black arrow) in the superficial dermis. Emigrant lymphocytes were seen in the dermis around vascular structures in all three tissue types (blue arrows). Hair follicles (d) consist of an internal (black arrow) and external root sheath (white arrow); a sebaceous gland (e) containing sebocytes (black arrows) filled with abundant lipid droplets and thin strands of cytoplasm; and an eccrine gland (f) with several cross sections of a coiled duct (black arrows) in unscarred skin. A low magnification view of a keloid scar (g) showing a flattened epidermis, thin strand-like collagen bundles in the SD and thick bundles of collagen arranged in a haphazard orientation within the actual KL in the reticular dermis. The central region of a KL (h) shows a high density of cells interspersed between the thick bundles of collagen (black arrows). The central region of another KL (I) shows thinner bundles of collagen arranged in a reticular pattern (black arrows). (Ep: epidermis; D: dermis). Scale bars: 100 μm (Original magnification a–f,h,i: 20×; g: 10×).

### Immunofluorescent staining

Tissue sections that were fixed in formalin for 48 h, paraffin-embedded and sectioned at a thickness of 4 μm were deparaffinized, rehydrated in graded ethanol solutions and blocked with phosphate-buffered saline (PBS)/2% (v/v) fetal bovine serum (FBS), prior to treatment with the Steptavidin/Biotin Blocking Kit (Vector Laboratories, Peterborough, UK). Sections were then incubated overnight with recombinant biotinylated HA-binding protein (5 μg/mL, Hokudo Co. Ltd, Sapporo, Japan) and rabbit antisera specific for TSG-6 [1 : 1000, RAH-1 (rabbit anti-human serum-1) raised against C-terminal peptide: TSTGNKNFLAGRFSHL], HC1 (1 : 1000, raised against N-terminal peptide: SATGRSKSSE), HC2 (1 : 1000, raised against C-terminal peptide: ESTPPPHVMRVE), or bikunin (1 : 1000, raised against N-terminal peptide: AVLPQEEEGS) diluted in PBS/2% (v/v) FBS.^23–25^ TSG-6 was also detected using affinity purified RAH-1 (see below) or an affinity purified polyclonal antibody raised against an internal peptide (ASVTAGGFQIK, referred to as ASV).^26^ Where appropriate the corresponding pre-immune sera, diluted 1 : 1000 in PBS/2% (v/v) FBS, were used as negative controls. Immunofluorescent detection was carried out using Alexafluor-488-streptavidin (Invitrogen, Oregon, USA) and Alexafluor-555-anti rabbit IgG (Invitrogen), diluted 1 : 500 in PBS/2% (v/v) FBS. Sections were mounted with Vectashield containing DAPI (4′,6-diamidino-2-phenylindole; Vector Laboratories) to allow visualization of cell nuclei.

**Figure 2:**
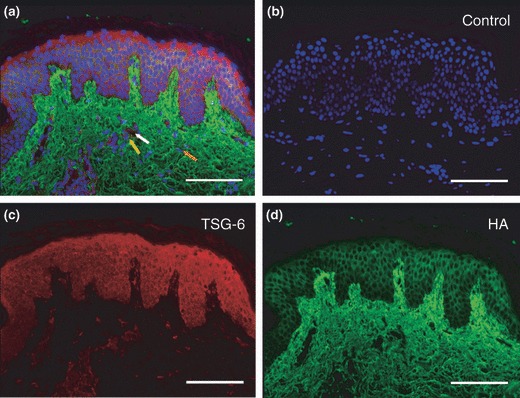
Immunofluorescent staining of unscarred skin. A combined image (a) is shown for HA (green), TSG-6 (red) and cell nuclei (blue) together with single filter images for TSG-6 (c), and HA (d). The negative control slide (b) was treated with the RAH-1 pre-immune serum. There was pericellular localization of HA in the epidermis and diffuse HA staining in the dermis. TSG-6 staining (a) was associated with keratinocytes in the epidermis and with fibroblasts (orange arrow), endothelial cells (white arrow) and perivascular lymphocytic cells (yellow arrow) in the dermis. Scale bars: 100 μm (Original magnification: 20×).

### Affinity purification of RAH-1

Tumour necrosis factor-stimulated gene-6 specific antibodies were purified from the RAH-1 antiserum by affinity chromatography, in which the TSG-6 peptide TSTGNKNFLAGRFSHL was covalently coupled to a Thiopropyl-Sepharose 6B gel (Mimotopes Pty. Ltd, Victoria, Australia). The purified antibody solution was concentrated by centrifugation in a Vivaspin 15R centrifugal concentrator (GE Healthcare UK Limited) for 30 min at 3000 ***g*** and 5 °C and its specificity for TSG-6 was confirmed by Western blot analysis with recombinant human TSG-6 protein.^12^

### Quantitative image analysis

Quantitative image analysis was carried out using WCIF ImageJ v1.40 (National Institute of Health, MA, USA). Staining intensity was measured by generating mean grey values from monochrome images of single antigen fluorescence in serial tissue sections. Statistical analysis of staining intensity with the independent samples *t*-test and one-way analysis of variance (anova) was carried out using spss 14.0 (SPSS Inc., Illinois, USA); *P* < 0.05 was considered to be significant.

## Results

### Histological analysis

Haematoxylin and eosin staining revealed that the most striking feature that distinguished unscarred skin (Fig. 1a) from both normal scars (Fig. 1b) and keloid scars (Fig. 1c) was the presence of an undulating dermal-epidermal junction and of skin appendages (i.e. hair follicles, sebaceous glands and eccrine sweat glands) (Figs 1d-f) in the dermis. The epidermis of normal scars and keloid scars were flatter than in unscarred skin, with less pronounced rete ridges and dermal papillae. The actual keloid lesion was localized to the reticular dermis and contained collagen arranged in a reticular pattern with an overall circular orientation forming characteristic ‘whorls’ or nodules (Figs 1g–i). Abnormally thick bundles of collagen, with haphazard orientation, were present within these whorls. Normal scars and keloid scars contained vascular structures in the dermis but skin appendages were absent. Perivascular lymphocytic infiltrates were seen in the dermis of unscarred skin, normal scars and in the dermis surrounding keloid lesions.

### Fluorescent localization of hyaluronan, TSG-6 and the polypeptide chains of IαI

**Figure 3:**
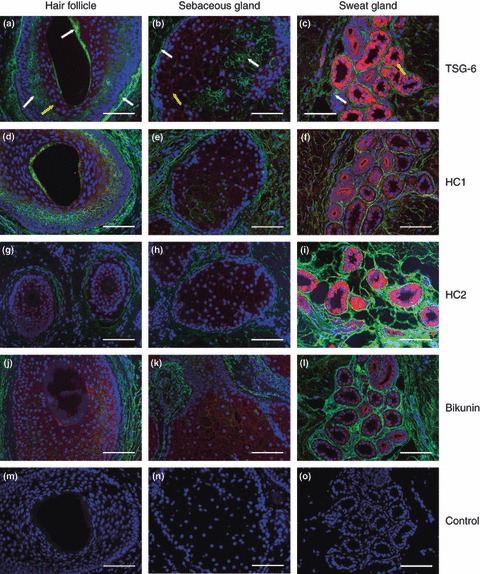
Immunofluorescent staining of skin appendages in unscarred skin. Combined images are shown for HA (green; a–l) with TSG-6 (red; a–c), HC1 (red; d–f), HC2 (red; g–i) or bikunin (red; j–l) and cell nuclei (blue). The negative control slides (m–o) were treated with the RAH-1 pre-immune serum; identical results were obtained for slides treated with the pre-immune sera corresponding to the antisera specific for HC1, HC2 and bikunin (not shown). The highest levels of HA staining (white arrows in a–c) were associated with the external aspects of hair follicle, intercellular space of external root sheaths and inner aspect of internal root sheaths (a), the external aspects and central parts of sebaceous glands (b) and the external aspects of eccrine sweat glands (c). TSG-6 staining (yellow arrows) was associated with the hair follicular cells (a), sebocytes (b) and secretory epithelial cells of eccrine sweat glands (c). Similar staining patterns were seen for HC1 (d–f), HC2 (g–i) and bikunin (j–l). Scale bars: 100 μm (Original magnification: 20×).

**Figure 4:**
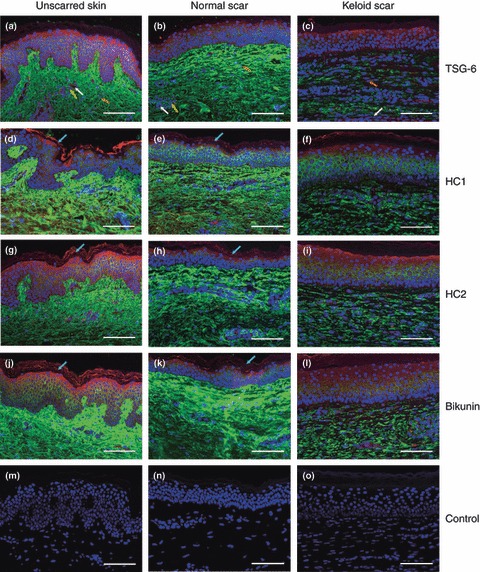
Immunofluorescent staining of unscarred skin, normal scars and keloid scars. Combined images are shown for HA (green; a–l) with TSG-6 (red; a–c), HC1 (red; d–f), HC2 (red; g–i) or bikunin (red; j–l) and cell nuclei (blue). The negative control slides (m–o) were treated with the RAH-1 pre-immune serum; identical results were obtained for slides treated with the pre-immune sera corresponding to the antisera specific for HC1, HC2 and bikunin (not shown). In unscarred skin (a,d,g,j) there was pericellular localisation of HA in the epidermis and diffuse HA staining in the dermis. In normal scars (b,e,h,k), and keloid scars (c,f,i,l) there was pericellular epidermal and strand-like dermal staining for HA. TSG-6 staining was associated with keratinocytes in the epidermis and with fibroblasts (orange arrows) and endothelial cells (white arrows) in unscarred skin (a) and normal scars (b) and keloid scars (c) as well as with perivascular lymphocytic cells (yellow arrows) in unscarred skin (a) and normal scars (b). Similar staining patterns were seen for HC1 (d–f), HC2 (g–i) and bikunin (j–l), except that there was increased staining in the keratin layers of unscarred skin and normal scars (cyan arrows). Scale bars: 100 μm (Original magnification: 20×).

**Figure 5:**
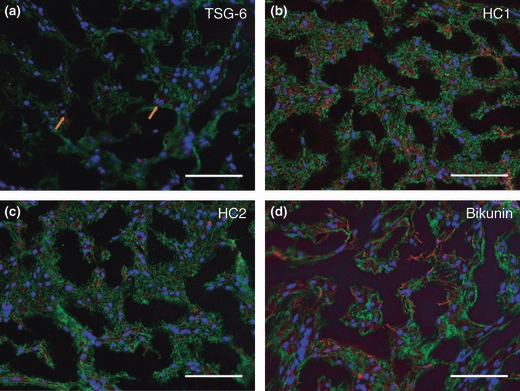
Immunofluorescent staining of keloid lesions within the reticular dermis. Combined images are shown for HA (green; a–d) with TSG-6 (red; a), HC1 (red; b), HC2 (red; c) or bikunin (red; d) and cell nuclei (blue). Reticular HA staining was seen between the thick bundles of collagen. Staining for TSG-6 (a), HC1 (b), HC2 (c) and bikunin (d) were localised to keloid fibroblasts (orange arrows). Scale bars: 100 μm (Original magnification: 20×).

This study has shown for the first time, the presence of TSG-6 and the IαI polypeptides HC1, HC2 and bikunin in human skin tissue. These molecules were present in unscarred skin, normal scars and keloid scars as described below. It should be noted that identical patterns of staining were seen for TSG-6 in experiments using the RAH-1 anti-serum (as illustrated in Figs 2–5) or the affinity purified RAH-1 or ASV polyclonal antibodies.

#### 

**Unscarred skin.** In the epidermis, HA staining (Fig. 2a,d) was predominantly pericellular, forming a reticular pattern around the keratinocytes. In the dermis, HA staining was variable in pattern and more intense than seen in the epidermis; the strongest HA staining was in the papillary dermis just below the epidermis. There was also intense HA staining surrounding blood vessels, hair follicles (Fig. 3a,d,g,j), sebaceous glands (Fig. 3b,e,h,k) and eccrine sweat glands (Fig. 3c,f,i,l). In particular there was pericellular HA staining around the epithelial cells of the external root sheath of the hair follicle and the sebocytes in sebaceous glands. There were no significant differences in HA staining in unscarred skin from regions adjacent to normal scars or keloid scars.

Tumour necrosis factor-stimulated gene-6 (Figs 2a,c and 4a), HC1, HC2 and bikunin (Fig. 4d,g,j) were seen to be associated with keratinocytes, melanocytes and Langerhans cells of the epidermis, and with fibroblasts, endothelial cells surrounding blood vessels and perivascular lymphocytic cells in the dermis. Within hair follicles, cells of the internal and external root sheaths stained for TSG-6 (Fig. 3a) and for all three IαI polypeptides (Fig. 3d,g,j). Staining for all four proteins was also seen to be associated with sebocytes within sebaceous glands (Fig. 3b,e,h,k) and the secretory epithelial cells lining eccrine sweat glands (Fig. 3c,f,i,l). There were no differences in TSG-6, HC1, HC2 or bikunin staining in unscarred skin adjacent to either normal scars or keloid scars.

#### 

**Normal scars.** Hyaluronan staining in the epidermis of normal scars showed a pericellular, reticular pattern similar to that seen in unscarred skin (Fig. 4b,e,h,k). In the dermis, there was extensive HA staining, where this was typically in a striated pattern oriented parallel to the flattened epidermis. The staining was most intense in the superficial dermis adjacent to the epidermis, and less so in the deeper dermis. As in unscarred skin, there was intense HA staining surrounding blood vessels. Staining patterns for TSG-6 (Fig. 4b) and the three polypeptide chains of IαI (Fig. 4e,h,k) in normal scars were similar to those observed in unscarred skin.

#### 

**Keloid scars.** The pattern of epidermal HA staining in keloid scars was similar to that seen in unscarred skin and normal scars (Fig. 4c,f,i,l). In the superficial dermis overlying the keloid lesion, HA staining had similar appearance to that, but was much less intense than, in the corresponding regions of unscarred skin and normal scars. Within the keloid lesion, HA staining was distinctly abnormal and occurred in a dense reticular pattern between the abnormally thick collagen bundles (Fig. 5).

The staining patterns for TSG-6 (Fig. 4c) and the IαI polypeptides (Fig. 4f,i,l) in the epidermis of keloid scars were similar to those seen in unscarred skin and normal scars. Within the dermis, all four molecules were found to be associated with fibroblasts, endothelial cells and lymphocytes. In the dermis overlying keloid lesions, spindle-shaped fibroblast-associated TSG-6 and IαI staining were seen (Figs. 4c,f,i,l), while the fibroblast-associated TSG-6 (Fig. 5a) and IαI (Fig. 5b,c,d) staining within keloid lesions was more rounded in appearance, consistent with the morphology of keloid fibroblasts.

### Quantitative differences in TSG-6 and HA staining intensities

**Figure 6:**
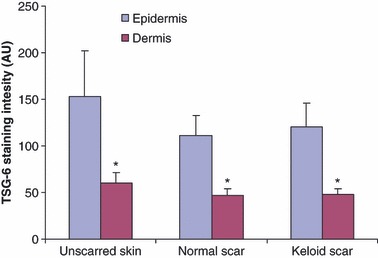
Quantification of TSG-6 staining intensity in unscarred skin (*n* = 3), normal scars and (*n* = 6) keloid scars (*n* = 7). Data are represented as mean staining intensity ± 2 x S.E.M. In all skin types there was significantly less TSG-6 detectable in the dermis compared to the epidermis (*); *P* = 0.029, *P* < 0.001 and *P* = 0.004 for unscarred skin, normal scars and keloid scars, respectively. There were no significant differences between the intensities of TSG-6 immunostaining either in the epidermis or in the dermis (immediately above lesions) when comparing unscarred skin, normal scars and keloid scars.

**Figure 7:**
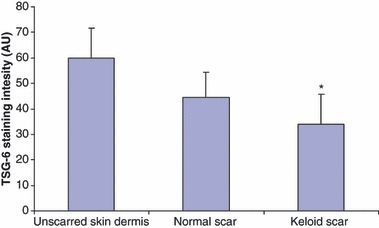
Quantification of TSG-6 staining intensity in unscarred skin dermis (*n* = 3), and in the centres of normal scars (*n* = 6) and keloid scars (*n* = 7). Data are represented as mean staining intensity ± 2 x S.E.M. TSG-6 immunostaining was significantly reduced in keloid lesions (*P* = 0.017), but not in normal scar lesions (*P* = 0.08), compared to unscarred skin dermis. * indicates *P* < 0.05.

Quantification on the basis of mean grey values revealed that there were significantly higher levels of TSG-6 immunostaining in the epidermis compared with the dermis of unscarred skin, normal scars and keloid scars (Fig. 6, Table 2). Comparison of TSG-6 staining intensities within the dermis (above scar lesions) or within the epidermis did not reveal any significant differences between unscarred skin, normal scars and keloid scars (Fig. 6). However, there was a significant reduction in the amount of TSG-6 detectable at the centre of keloid lesions compared with unscarred skin dermis (*P* = 0.017; Fig. 7)**.**

**Table 2:** Mean TSG-6 staining intensity levels (AU) of unscarred skin, normal scars and keloid scars

Position	Unscarred skin (AU)	Normal scar (AU)	Keloid scar (AU)
Epidermis	136.55	110.85	124.52
Dermis	56.43	46.86	49.74
Mid-scar	NA	44.50	32.10

TSG-6; tumour necrosis factor-stimulated gene-6.

**Figure 8:**
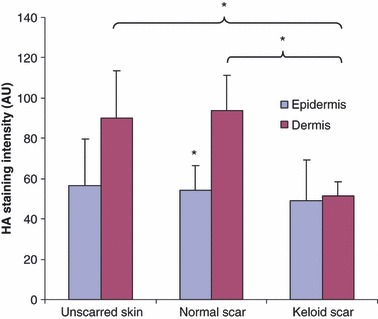
Quantification of HA staining intensity in unscarred skin (*n* = 3), normal scars (*n* = 6) and keloid scars (*n* = 7). Data are represented as mean staining intensity ± 2 x S.E.M. There was significantly less HA (*) detectable in the dermis compared to the epidermis of normal scars (*P* = 0.004). The reduced HA staining in unscarred skin epidermis compared with unscarred skin dermis staining was not significant (*P* = 0.086). There was no significant difference between HA staining intensities of keloid epidermis and keloid dermis. Keloid dermis showed significantly reduced staining for HA (*) compared with unscarred skin (*P* = 0.04) and normal scar dermis (*P* = 0.003).

Hyaluronan staining intensity was higher in the dermis compared with the epidermis in unscarred skin and normal scars (Fig. 8), but this difference was statistically significant only in the latter case (*P* = 0.004). Within keloid scars, HA staining intensities were essentially the same in the dermis and epidermis. Keloid dermis showed significantly reduced staining for HA compared with unscarred skin dermis (*P* = 0.04) and normal scar dermis (*P* = 0.003; Fig. 8). There were also small, but significant, reductions in HA staining intensity within keloid lesions compared with normal scar lesions (*P* = 0.031) and unscarred skin dermis (*P* = 0.017) (data not shown).

## Discussion

This study has shown for the first time, the presence of TSG-6 and the IαI polypeptides (HC1, HC2 and bikunin) within the skin. TSG-6 was originally described in tumour necrosis factor-α-stimulated foreskin fibroblasts^27^ and its expression is known to be upregulated in response to various inflammatory mediators and growth factors.^14,15^ Although TSG-6 is associated mostly with pathological conditions (e.g. osteoarthritis, rheumatoid arthritis and asthma),^25,28^ it also has an important physiological role in catalyzing the formation of HC•HA complexes that are essential for cumulus matrix expansion in ovarian follicles, where TSG-6 expression occurs in response to ovulatory stimuli.^21,29–31^ These complexes form at sites of inflammation where there is upregulation of TSG-6 and HA expression and ingress of IαI from serum.^18,20^

The observation of TSG-6 staining associated with cells of the skin (keratinocytes, melanocytes and Langerhans cells in the epidermis, as well as fibroblasts, endothelial cells and perivascular lymphocytic cells in the dermis) suggests that these cells are synthesizing TSG-6 locally. The epidermis is a specialized structure that provides a continuously regenerating barrier to environmental insults and prevents loss of fluids. This is achieved through the terminal differentiation of keratinocytes, in a process termed ‘cornification’, which leads to keratin deposition on the superficial surface of the skin.^32^ Hair follicles and sebaceous glands are modified epithelial structures, each lined with a continually regenerating layer of terminally differentiating epithelial cells, that mediate deposition of keratin and sebum respectively.^33^ Expression of TSG-6 in the epidermis, hair follicles and sebaceous glands may be a feature of the continuous turnover of cells and ECM remodelling that is associated with these key physiological functions; recent evidence suggests that TSG-6 may have a role in the regulation of cell differentiation.^26,34^ The relevance of TSG-6 production by endothelial cells and perivascular lymphocytic cells in the skin remains to be determined. However, this could reflect its importance in the regulation of acute inflammation (e.g. by inhibiting neutrophil migration).^35,36^

The observation of TSG-6 immunostaining within unscarred skin might suggest a role for TSG-6 in maintaining the architecture of this tissue. Any reduction in this activity could potentially contribute to the processes of scarring. In this regard, a recent study revealed that secretion of TSG-6 by intravenously infused human multipotent stromal cells is the key factor in improving cardiac function in a mouse model of myocardial infarction (MI).^37^ TSG-6 is found to reduce the extent of proteolytic damage and fibrotic scarring in heart tissue, which is attributed to suppression of pro-inflammatory protease activity (e.g. plasmin and matrix metalloproteinases). Here we have observed a small, but significant, decrease in the amount of TSG-6 detectable in the centre of keloid lesions compared with the dermis of unscarred skin. This may be a consequence of reduced protein expression *per se* or a reflection of altered tissue structure and/or cell content.

Cell-associated immunostaining for all three IαI polypeptides, similar to that seen for TSG-6, might also be indicative of local expression in the skin. Although IαI was previously thought to be synthesized only in the liver and distributed to different tissues through serum, there is recent evidence of local expression of its individual chains in some articular chondrocytes,^24^ airway epithelial and submucosal gland cells of smokers.^25^ The abundant presence, and close proximity to each other, of TSG-6, IαI and HA in unscarred skin opens up the possibility that TSG-6-mediated HC•HA formation and hence HA cross-linking might occur in the skin, where this might contribute to skin physiology and homeostasis.

The localization patterns of HA in unscarred skin, normal scars and keloid scars seen in this study are similar to those reported previously.^38,39^ The HA staining associated with skin appendages shown here is also similar to that described previously.^40,41^ There is good evidence for the involvement of HA in the reduction of scarring during skin repair. For example, keloid-derived fibroblasts have been found to accumulate lower levels of HA compared to normal scar or normal skin fibroblasts when cultured *in vitro*,^38^ which is consistent with this current study. In contrast, another study showed an increase in the incorporation of radiolabelled precursors into HA in cultured keloid fibroblasts compared with normal skin fibroblasts.^42^ As addressed by Meyer *et al.*,^38^ this difference between HA accumulation and synthesis may be attributed to differences in actual HA turnover.

In this study, HA staining was seen to be aligned parallel to fibroblasts with abnormal matrix deposition patterns in keloid scars. Previous work has revealed that HA deposition can influence the orientation and shape of fibroblasts as well as promoting their migration and deposition of collagen (the major component of mature scars).^43^ A recent study has revealed that HA-synthase 2 (HAS2)-dependent HA synthesis and expression of TSG-6 by fibroblasts is essential for TGF-β1-mediated fibroblast to myofibroblast differentiation.^34^ The correct organization of the HA matrix in the pericellular coat is of particular importance in this process, where this is probably dependent on TSG-6. The abnormal HA deposition patterns seen in keloid lesions in this study may play a role in influencing keloid fibroblast morphology and behaviour, resulting in the distinct histological pathology. The reduced TSG-6 expression in keloid lesions may contribute to the abnormal HA matrix.

This study has shown that TSG-6 and IαI are present in the skin, together with HA, where high levels of these proteins in the epidermis and skin appendages suggest that TSG-6-mediated HC•HA formation could be important in the maintenance of these structures. TSG-6 is encoded within chromosome 2q23.3 – i.e. a region of the genome reported to contain a susceptibility locus for keloid scarring. The results of this study indicated that there is a small reduction in TSG-6 protein levels within keloid lesions compared with unscarred skin dermis. This suggests that loss of TSG-6 function might be a contributory factor to the abnormal scarring process underlying keloid formation; further work is needed to investigate this.
